# HLA-C polymorphisms with susceptibility and protection against acute leukemia in a Moroccan population

**DOI:** 10.3389/fimmu.2026.1869016

**Published:** 2026-07-31

**Authors:** Khalid Laaziri, Ikram Brahim, Raja Hazime, Fatima Ezzahra Lahlimi, Ouadii Abakarim, Illias Tazi, Jalila EL Bakkouri, Hanae Ettayebi, Mounia Ammara, Abdelmajid Zyad, El Mostafa Mtairag, Brahim Admou

**Affiliations:** 1 Laboratory of Integrative Biology, Faculty of Science, Ain Chock, Hassan II University, Casablanca, Morocco; 2 Laboratory of Immunology and HLA, Center of Clinical Research, Mohammed VI University Hospital, Marrakech, Morocco; 3 Bioscience Research Laboratory, Faculty of Medicine and Pharmacy, Cadi Ayyad University, Marrakech, Morocco; 4 Department of Clinical Hematology and Bone Marrow Transplantation, Mohammed VI University Hospital, Marrakech, Morocco; 5 Laboratory of Immunology of University Hospital Ibn Rochd, Casablanca, Morocco; 6 Laboratory of Materials, Nanotechnologies and Environment Faculty of Sciences, Rabat Mohammed V University, Rabat, Morocco; 7 Laboratory of Agro-Industrial and Medical Biotechnology, Team of Experimental Oncology and Natural Substances, Cellular and Molecular Immuno-Pharmacology, Sultan Moulay Slimane University, Faculty of Sciences and Techniques, Beni Mellal, Morocco

**Keywords:** acute lymphoid leukemia, acute myeloid leukemia, genotypes, HLA-C polymorphism, Morocco

## Abstract

**Background:**

Acute leukemia (AL) is a malignant hematological disorder, with two main subtypes: acute myeloid leukemia (AML) and acute lymphoblastic leukemia (ALL). The incidence rate of AML and ALL is estimated at approximately 2.7 and 1.5 per 100,000 people, respectively, with an overall mortality rate of approximately 23.6%. This study aimed to investigate the association between human leukocyte antigen (HLA)-C genotypes and AML and ALL in a Moroccan population.

**Patients and methods:**

A cross-sectional and comparative study was conducted on 100 patients with AL including 43 cases of AML and 57 cases of ALL, compared to 120 healthy individuals as controls. HLA-C genotyping was performed in the two groups, using the PCR-SSO (polymerase chain reaction–sequence-specific oligonucleotide)-based technique.

**Results:**

The mean age of the patients was 32 ± 13 (2–62), with a sex ratio of 1.22 (55 male and 45 female patients). In patients with ALL categories, we observed a significantly higher frequency of HLA-C*12 [*p* = 0.001; odds ratio (OR) = 7.58] allele groups. In the AML category, significant associations were observed between AML and the HLA-C*12 (*p* = 0.0001; OR = 10.35) and HLA-C*08 (*p* = 0.005; OR = 8.92) allele groups.

**Conclusion:**

Our findings show a strong association between HLA-C*12 and HLA-C*08 allele groups and AL, including both ALL and AML, which gives evidence of a predisposing effect. This highlights the influence of HLA polymorphism in the occurrence or resistance of AL in our population.

## 1 Introduction

Acute leukemia (AL) is a hematological malignancy characterized by two primary subtypes: acute myeloid leukemia (AML), which constitutes over 80% of cases, and acute lymphoblastic leukemia (ALL), accounting for roughly 15% of cases. The incidence rates of AML and ALL are estimated to be 2.7 and 1.5 per 100,000 individuals, respectively, with the highest mortality rate among all malignancies, estimated at 23.6% (1). Although treatments for AL have significantly improved in recent years, the disease continues to progress swiftly and is generally lethal.

The World Health Organization (WHO) categorizes ALL as B-cell precursor acute lymphoblastic leukemia (BCP-ALL) and T-cell acute lymphoblastic leukemia (T-ALL). AML is categorized into six classifications: AML with recurrent genetic abnormalities; AML associated with myelodysplastic syndromes (MRC); therapy-related myeloid neoplasms (t-MN); AML not otherwise specified (NOS); myeloid sarcoma; and myeloid proliferations linked to Down syndrome (DS) (2).

ALL is a rapidly proliferating malignancy that converts white blood cells (WBCs) into aberrant and excessive lymphoblasts, hence compromising the immune system and leading to mortality. AML is a swiftly proliferating cancer that significantly compromises bone marrow and myeloid WBCs, necessitating prompt diagnosis and suitable intervention (3). The principal approaches employed to describe all kinds of AL include morphological assessment, immunophenotypic analysis, molecular genetic testing, and cytogenetic evaluation (4).

Human leukocyte antigen (HLA)-C has garnered considerable interest in biomedical research owing to its role in viral and cancer defense, as well as its association with autoimmune diseases, preeclampsia, and allograft rejection (5, 6).

A specific decrease in HLA-C expression on juvenile ALL blasts was observed, indicating that these modifications in tumor surface markers may influence immune recognition and the progression of the disease (7).

Conversely, patients with hematological malignancies, such as ALL and AML, exhibit functional impairments in natural killer (NK) cells, characterized by diminished expression of NK activating receptors, which correlates with reduced NK cell activity in individuals with AML and ALL. NK cells attack malignant cells when signals from activating receptors surpass the inhibitory signals from receptors that engage major histocompatibility complex (MHC) class I. It is proposed that the activation of receptors such as DNAM-1, NKp46, and NKp30 facilitates NK cell identification of blasts in AL (8).

Similarly, it has been shown that extremely low expression of HLA-C within B-cell populations was significantly associated with a subgroup of leukemias exhibiting a CD19^+^CD45^−^ phenotype. Conversely, B-cell populations with low HLA-C levels vanished after full remission, which supports the disappearance of leukemic blast populations (7).

Furthermore, the HLA-C gene does not merely play a predisposing or protective role with respect to AL; its influence varies depending on specific HLA-C alleles, their expression levels, and their interaction with NK cell KIR receptors. Certain HLA-C allele groups or genotypes, such as C2, C3, or Cw3/Cw4, are associated with an increased risk of leukemia and relapse, whereas homozygosity for C1 and certain alleles, such as C*01, are likely protective. These effects are primarily due to the modulation of NK cell maturation and activation, as well as tumor immune evasion, including downregulation of HLA-C on leukemic cells (9–11).

This study aimed to investigate the relationship between the polymorphism of HLA-C allelic groups and genotypes and myeloid and lymphoblastic AL in Moroccan pediatric and adult populations.

## 2 Patients and methods

### 2.1 Study design

The patient group consisted of 100 individuals presenting with AL, including 43 cases of AML and 57 cases of ALL. Among patients with AML, AML-2 was the predominant subtype (*n* = 27; 62.79%), while in the ALL group, B-cell precursor ALL (BCP-ALL) represented most cases (*n* = 43; 75.43%). A large percentage of patients had chemotherapy, and they were all candidates for bone marrow transplants. AL subtypes were classified according to the French–American–British (FAB) classification system (12).

The control group included 120 healthy individuals. Patients and controls were recruited during the decade 2014–2025 from different areas of southern Morocco, including Marrakech-Safi, Souss-Massa, Beni-Mellal-Khénifra, Guelmim-Oued Noun, and Drâa-Tafilalet. HLA-C genotype frequencies were used to assess Hardy–Weinberg equilibrium (HWE) in the control group. The control population was genetically representative, and no substantial genotyping bias was found because there was no significant divergence from HWE (χ² = 79.13, df = 78, *p* = 0.44).

The diagnosis of AL was validated using complete blood count analysis, peripheral blood smear evaluation, and bone marrow testing. Inclusion criteria were formulated after a comprehensive examination of patients’ medical records in conjunction with the pediatric and adult hematology departments. Patients were chosen among those submitted to the HLA laboratory of the University Hospital as candidates for hematopoietic stem cell transplantation (HSCT). The control group consisted of healthy adults chosen from prospective related donors. Individuals with hematological malignancies, except AML or ALL, were omitted from the study.

### 2.2 Ethical considerations

Patient and control samples analyzed in this study were obtained during the routine activity of the laboratory. Sociodemographic and clinical information was anonymously extracted from the laboratory database under the supervision of the laboratory manager. Patients and controls had previously been clearly informed of the purpose of the investigations initiated by clinicians as part of their general care, with the aim of performing a hematopoietic cell transplant from a related donor.

### 2.3 Sample collection and processing

Peripheral venous blood was collected using two 5-mL tubes containing ethylenediaminetetraacetic acid (EDTA) as an anticoagulant according to a rigorously defined protocol designed for HLA typing. Upon collection, the samples were packaged in specific transportation bags and immediately transferred to the laboratory to maintain sample integrity during transit, which is critical to prevent degradation and to ensure the preservation of the cellular components necessary for accurate HLA typing.

### 2.4 DNA extraction

Genomic DNA extraction from buffy coat (float of peripheral venous blood was collected using two 5-mL tubes of EDTA and centrifuged for 10 min at 5,000 *g*) was executed using the QIAmp DNA Mini kit (Qiagen, Hilden, Germany) following a multi-step protocol. Briefly, this process began with cell lysis, where the buffy coat was treated with a lysis buffer containing chaotropic salts to disrupt cell membranes and release genomic DNA. The lysate was then applied to a silica-based column, where the genomic DNA was selectively bound to the silica membrane, allowing for impurities to be washed away. A series of ethanol-based wash buffers was used to cleanse the DNA, ensuring the removal of salts, metabolites, and other contaminants. Finally, the purified DNA was eluted from the membrane using an elution buffer, carefully releasing the DNA without causing damage.

The resulting DNA was quantitatively and qualitatively assessed using a NanoDrop™ 2000/2000c Spectrophotometer (Thermo Scientific™, Waltham, Massachusetts, MA, USA), which measured DNA concentration and purity, crucial for downstream molecular analyses.

### 2.5 HLA-C typing

HLA-C typing was performed utilizing two separate polymerase chain reaction (PCR) platforms, employing sequence-specific oligonucleotide (SSO) PCR, supplied by Immucor™ (LIFECODES^®^ HLA-SSO Typing, Massachusetts, Waltham, MA, USA) and OneLambda (Thermo Fisher Scientific, LabType™, Waltham, MA, USA). This method involves PCR amplification, followed by the hybridization of the amplified products to beads coated with oligonucleotide probes unique to known HLA sequences. The hybridization patterns were further evaluated using Luminex technology to detect the fluorescent signals of the microspheres. The color coding of each microsphere corresponds to a specific HLA probe, while the fluorescence intensity reflects the hybridization strength, facilitating the identification of the HLA-C locus and specificities present in the sample.

The analysis of the supplied PCR typing data was conducted using Fusion 4.4^®^ (One Lambda) or MATCH IT!^®^ (Immucor) software, version 4.1.0 (Norcross, GA, USA), which incorporates fluorescence intensity data from Luminex xMAP technology. This software employs a database of established allele sequences to correlate with the observed probe fluorescence patterns, delivering an extensive profiling of HLA-C and specificities for patients and controls.

### 2.6 Statistical analysis

Data analysis was performed with the IBM SPSS Statistics (IBM, Armonk, NY, USA) program, and the χ^2^ test was used to evaluate differences in HLA-C allele group frequencies between patients and controls. We also used Jamovi v.1.2 (The Jamovi Project, Sidney, Australia) to make the graphs of each significant allelic group. Results were considered statistically significant when the *p*-value ≤ 0.05. However, we applied a multiple-test correction using the Bonferroni method. In order to compare the variability of HLA protective allele groups and genotypes between patients and controls, R version 4.1.0 was applied. The prevalence was counted according to diagnostic information, and the association strength was estimated by odds ratio (OR) and 95% confidence interval (CI).

## 3 Results

### 3.1 Patient characteristics

As indicated in **Table 1**, the mean age of all patients was 32 ± 13 years (range: 2–62 years) compared to 35.5 ± 16.5 years in the control group (range: 4–52 years). This study revealed a male predominance of 53.77%, resulting in a male-to-female sex ratio of 1.2, compared to 0.8 in the control group, as shown in patients’ characteristics in **Table 1**.

**Table 1:** Demographic and clinical characteristics of patients.

Characteristics	Patients with acute leukemia *n* (%)
Total patients	100
Mean age (ranges)	32 ± 13 (2–62 years)
Gender	
Female	45 (46.22%)
Male	55 (53.77%)
Patients’ race	
White	85 (85.00%)
Black	15 (15.00%)
Categories of AML	43
AML 0	0 (0.00%)
AML 1	0 (0.00%)
AML 2	27 (62.79%)
AML 3	7 (16.27%)
AML 4	6 (13.95%)
AML 5	0 (0.00%)
AML 6	0 (0.00%)
AML 7	0 (0.00%)
Unclassified	2 (4.65%)
Categories of ALL	57
BCP-ALL	43 (75.44%)
TCP-ALL	14 (24.56%)

The examination of the association between HLA-C and blood types showed no significant relation. No significant differences were observed between male and female patients regarding the frequencies of various HLA alleles.

### 3.2 Assessment of the frequency of HLA-C allelic groups in patients with ALL

**Figure 1:**
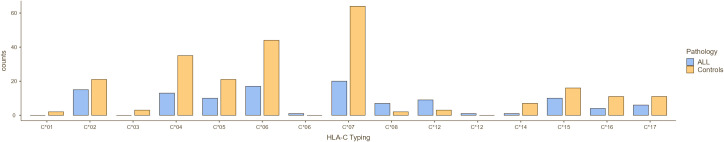
Comparative frequency of HLA-C between patients with ALL and controls.

HLA-C typing was performed on 57 patients with ALL and 120 control individuals. The average age of the patients with ALL was 19 years, with a range of 2 to 36 years. A total of 13 HLA-C allelic groups were analyzed in both patients and controls, with HLA-C07 being the most frequent at 17.5%. Compared to the control group, adult patients with AML showed a higher frequency of the HLA-C allelic groups C*12 (*p* = 0.001, OR: 7.58) as designated in **Table 2** and **Figure 1** below.

**Table 2:** Comparative frequency of HLA-C allele groups in patients with ALL and controls.

HLA-C allele groups	ALL (2*n* = 114) *n* (%)	Controls (2*n* = 240) *n* (%)	OR (95%CI)	*p*-value	Corrected *p*-value (Bonferroni method)	Power (%)
C*01	0 (0.0%)	2 (0.8%)	0.42 (0.02–8.78)	1.000	1.000	31
C*02	15 (13.2%)	21 (8.8%)	1.59 (0.79–3.18)	0.258	1.000	24
C*03	0 (8.8%)	3 (1.3%)	1.41 (0.07–28.3)	0.56	1.000	6
C*04	13 (11.4%)	35 (14.6%)	0.75 (0.38–1.47)	0.507	1.000	11
C*05	10 (8.8%)	21 (8.8%)	1.00 (0.46–2.16)	1.000	1.000	5
C*06	18 (15.78%)	44 (18.3%)	0.84 (0.46–1.55)	0.654	1.000	9
C*07	20 (17.5%)	64 (26.7%)	0.58 (0.33–1.03)	0.062	0.806	46
C*08	**7 (6.1%)**	**2 (0.8%)**	**7.76 (1.61**–**37.4)**	**0.006**	**0.078**	**80**
C*12	**10 (8.8%)**	**3 (1.3%)**	**7.58 (2.02**–**28.4)**	**0.001**	**0.013**	**91**
C*14	1 (0.9%)	7 (2.9%)	0.30 (0.04–2.40)	0.445	1.000	18
C*15	10 (8.8%)	16 (6.7%)	1.36 (0.59–3.15)	0.515	1.000	14
C*16	4 (3.5%)	11 (4.6%)	0.76 (0.24–2.40)	0.782	1.000	8
C*17	6 (5.3%)	11 (4.6%)	1.16 (0.42–3.21)	0.793	1.000	6

OR, odds ratio; CI, confidence interval, which describes the odds of cases being HLA allele carriers to the odds of controls being HLA allele carriers. Specifically, OR = 1 indicates that the factor has no impact on the occurrence of the disease, OR >1 indicates that the factor is a risk factor, and OR <1 indicates that the factor is protective.

The frequencies of the HLA-C allelic groups are illustrated in **Figure 1** below, which clearly shows the prevalence of the HLA-C*12 and HLA-C*08 allelic groups in patients with ALL compared with the control group.

### 3.3 Frequency determination of HLA-C genotypes in patients with ALL

The frequencies of HLA-C genotypes were compared between 57 patients with ALL and 120 healthy controls. The most frequent genotype among patients with ALL was HLA-C02/C07, observed in 8.8% of individuals. However, none of the HLA-C genotypes was substantially linked with ALL susceptibility after Bonferroni correction for multiple testing (shown in **Table 3**).

**Table 3:** Comparative frequency of HLA-C genotypes in patients with ALL and controls.

HLA-C genotypes	ALL (*n* = 57) *n* (%)	Controls (*n* = 120) *n* (%)	OR (95% CI)	*p*-value	Corrected *p*-value (Bonferroni method)	Power (%)
C*02/C*04	1 (1.8%)	3 (2.5%)	0.69 (0.07–6.94)	1.000	1.000	6
C*02/C*05	2 (3.5%)	4 (3.3%)	1.05 (0.19–5.74)	1.000	1.000	5
C*02/C*06	2 (3.5%)	6 (5.0%)	0.69 (0.13–3.59)	1.000	1.000	5
C*02/C*07	5 (8.8%)	6 (5.0%)	1.83 (0.52–6.38)	0.335	1.000	14
C*04/C*05	2 (3.5%)	3 (2.5%)	1.41 (0.23–8.57)	0.658	1.000	6
C*04/C*06	1 (1.8%)	6 (5.0%)	0.34 (0.04–2.82)	0.431	1.000	11
C*04/C*07	0 (0.0%)	12 (10.0%)	0.07 (0.004–1.20)	0.010	0.200	72
C*05/C*12	3 (5.3%)	0 (0.0%)	15.2 (0.78–296)	0.04	0.800	65
C*04/C*15	4 (7.0%)	2 (1.7%)	4.46 (0.78–25.5)	0.086	1.000	45
C*05/C*06	0 (0.0%)	6 (5.0%)	0.17 (0.01–2.93)	0.179	1.000	18
C*05/C*07	0 (0.0%)	4 (3.3%)	0.25 (0.01–4.71)	0.307	1.000	10
C*06/C*06	3 (5.3%)	1 (0.8%)	6.65 (0.67–65.7)	0.099	1.000	40
C*06/C*07	3 (5.3%)	9 (7.5%)	0.69 (0.18–2.61)	0.754	1.000	7
C*06/C*08	3 (5.3%)	1 (0.8%)	6.65 (0.67–65.7)	0.099	1.000	40
C*06/C*14	0 (0.0%)	3 (2.5%)	0.34 (0.02–6.47)	0.552	1.000	5
C*06/C*15	1 (1.8%)	3 (0.8%)	0.70 (0.07–6.94)	1.000	1.000	5
C*06/C*16	0 (0.0%)	5 (4.2%)	0.19 (0.01–3.20)	0.177	1.000	17
C*07/C*15	2 (3.5%)	8 (6.7%)	0.51 (0.10–2.52)	0.504	1.000	9
C*07/C*17	2 (3.5%)	3 (2.5%)	1.41 (0.23–8.57)	0.658	1.000	6
C*16/C*17	0 (0.0%)	3 (2.5%)	0.34 (0.02–6.47)	0.552	1.000	5

OR, odds ratio; CI, confidence interval, which describes the odds of cases being HLA allele carriers to the odds of controls being HLA allele carriers. Specifically, OR = 1 indicates that the factor has no impact on the occurrence of the disease, OR >1 indicates that the factor is a risk factor, and OR <1 indicates that the factor is protective.

The results did not include the frequency of HLA-C genotypes less than three in both groups.

### 3.4 Frequency determination of HLA-C allelic groups in ALL subgroups

Based on the statistical significance of the ALL subgroups, we will examine only the BCP-ALL subtype, given the sample size and the statistically significant results found.

#### 3.4.1 Frequency determination of HLA-C allelic groups in patients with BCP-ALL

We have analyzed the HLA-C typing for 43 patients with BCP-ALL and 120 control individuals. The mean age of the patients with BCP-ALL was 18 years (ranging from 3 to 33 years). In total, 13 HLA-C allelic groups were examined; the frequency of HLA-C*06 was the highest, with 15.11%.

**Figure 2:**
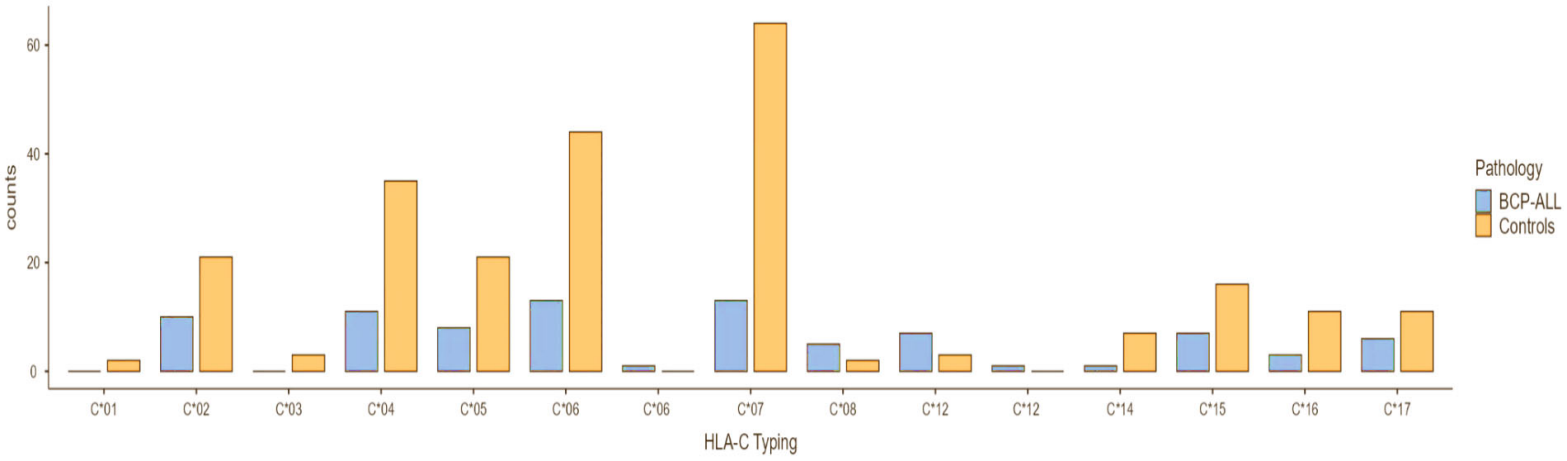
Comparative frequency of HLA-C between patients with BCP-ALL and controls.

In comparison to the control group, HLA-C*12 (*p* = 0.001, OR: 8.19) showed increased frequency in patients with BCP-ALL, as stated in **Table 4** and **Figure 2** below.

**Table 4:** Comparative frequency of HLA-C allele groups in patients with BCP-ALL and controls.

HLA-C allele groups	BCP-ALL (2*n* = 86) *n* (%)	Controls (2*n* = 240) *n* (%)	OR (95% CI)	*p*-value	Corrected *p*-value (Bonferroni method)	Power (%)
C*01	0 (0.0%)	2 (0.8%)	0.55 (0.03–11.2)	1.000	1.000	5
C*02	10 (11.6%)	21 (8.8%)	1.36 (0.62–2.98)	0.520	1.000	13
C*03	0 (0.0%)	3 (1.3%)	0.42 (0.02–8.37)	0.569	1.000	5
C*04	11 (12.8%)	35 (14.6%)	0.86 (0.41–1.80)	0.857	1.000	7
C*05	8 (9.3%)	21 (8.8%)	1.06 (0.44–2.54)	0.829	1.000	5
C*06	14 (15.11%)	44 (18.3%)	0.78 (0.41–1.49)	0.620	1.000	9
C*07	**13 (15.1%)**	**64 (26.7%)**	**0.49 (0.25**–**0.97)**	0.04	0.520	58
C*08	**5 (5.8%)**	**2 (0.8%)**	**7.25 (1.35**–**38.9)**	0.02	0.260	73
C*12	**8 (9.30%)**	**3 (1.3%)**	**8.19 (2.04**–**32.8)**	0.001	**0.013**	88
C*14	1 (1.2%)	7 (2.9%)	0.75 (0.21–2.63)	0.686	1.000	6
C*15	7 (8.1%)	16 (6.7%)	1.57 (0.54–4.55)	0.629	1.000	15
C*16	3 (3.5%)	11 (4.6%)	0.55 (0.03–11.2)	1.000	1.000	5
C*17	6 (7.0%)	11 (4.6%)	1.36 (0.62–2.98)	0.402	1.000	11

OR, odds ratio; CI, confidence interval, which describes the odds of cases being HLA allele carriers to the odds of controls being HLA allele carriers. Specifically, OR = 1 indicates that the factor has no impact on the occurrence of the disease, OR >1 indicates that the factor is a risk factor, and OR <1 indicates that the factor is protective.

**Figure 2** illustrates the prevalence of HLA-C allelic groups C*08 and C*12 in patients with BCP-ALL in comparison to controls.

#### 3.4.2 Frequency determination of HLA-C genotypes in patients with BCP-ALL

We analyzed the HLA-C genotypes of patients with BCP-ALL and 120 healthy individuals; C*05/C*12 and C*06/C*08 were the most frequent, with 7.0%, as detailed in **Table 5** below.

**Table 5:** Comparative frequency of HLA-C genotypes in patients with BCP-ALL and controls.

HLA-C genotypes	BCP-ALL (*n* = 43) *n* (%)	Controls (*n* = 120) *n* (%)	OR (95% CI)	*p-*value	Corrected *p*-value (Bonferroni method)	Power (%)
C*02/C*04	2 (4.7%)	3 (2.5%)	1.92 (0.30–12.2)	0.64	1.000	8
C*02/C*05	1 (2.3%)	4 (3.3%)	0.68 (0.07–6.72)	1.00	1.000	5
C*02/C*06	2 (4.7%)	6 (5.0%)	0.93 (0.18–4.73)	1.00	1.000	5
C*02/C*07	2 (4.7%)	6 (5.0%)	0.93 (0.18–4.73)	1.00	1.000	5
C*04/C*05	2 (4.7%)	3 (2.5%)	1.92 (0.30–12.2)	0.64	1.000	8
C*04/C*06	1 (2.3%)	6 (5.0%)	0.45 (0.05–3.85)	0.45	1.000	9
C*04/C*07	**0 (0.0%)**	**12 (10.0%)**	**0.09 (0.005**–**1.53)**	**0.03**	0.60	68
C*04/C*15	3 (7.0%)	2 (1.7%)	4.47 (0.71–28.1)	0.11	1.000	41
C*05/C*06	0 (0.0%)	6 (5.0%)	0.19 (0.01–3.32)	0.17	1.000	15
C*05/C*07	1 (2.3%)	4 (3.3%)	0.68 (0.07–6.72)	1.00	1.000	5
C*05/C*12	3 (7.0%)	0 (0.0%)	21.7 (1.08–435)	0.02	0.40	62
C*06/C*07	1 (2.3%)	9 (7.5%)	0.29 (0.04–2.29)	0.29	1.000	8
C*06/C*08	3 (7.0%)	1 (0.8%)	8.95 (0.88–91.2)	0.09	1.000	38
C*06/C*14	0 (0.0%)	3 (2.5%)	0.41 (0.02–7.76)	0.56	1.000	5
C*06/C*15	1 (2.3%)	3 (2.5%)	0.93 (0.09–9.33)	1.00	1.000	5
C*06/C*16	0 (0.0%)	5 (4.2%)	0.22 (0.01–3.89)	0.32	1.000	14
C*07/C*07	1 (2.3%)	9 (7.5%)	0.29 (0.04–2.29)	0.29	1.000	8
C*07/C*15	2 (4.7%)	8 (6.7%)	0.68 (0.14–3.39)	1.00	1.000	9
C*07/C*17	2 (4.7%)	3 (2.5%)	1.92 (0.30–12.2)	0.64	1.000	8
C*16/C*17	0 (0.0%)	3 (2.5%)	0.41 (0.02–7.76)	0.56	1.000	5

OR, odds ratio; CI, confidence interval, which describes the odds of cases being HLA allele carriers to the odds of controls being HLA allele carriers. Specifically, OR = 1 indicates that the factor has no impact on the occurrence of the disease, OR >1 indicates that the factor is a risk factor, and OR <1 indicates that the factor is protective.

The results did not include the frequency of HLA-C genotypes less than three in both groups.

### 3.5 Frequency of HLA-C allelic groups in the AML population

**Figure 3:**
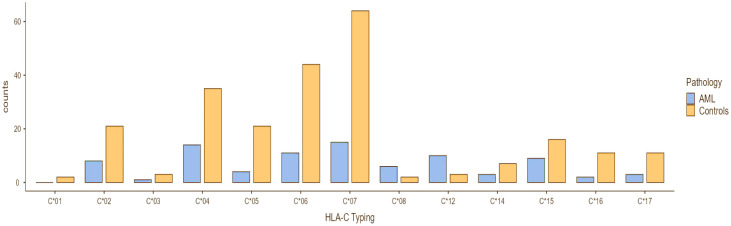
Comparative frequency of HLA-C between patients with AML and controls.

We analyzed the HLA-C allelic groups analyzed in 43 patients with AML and 120 healthy individuals. The average age of the patients with AML was 34.5 years, with a range of 7 to 62 years. The frequency of HLA-C*07 was the highest, with 17.4%, but only HLA-C*12 (*p* = 0.0001, OR: 10.35) showed a significant increased frequency in patients with AML compared to controls as specified in **Table 6** and **Figure 3** below:

**Table 6:** Comparative frequency of HLA-C allele groups between patients with AML and controls.

HLA-C allele groups	AML (2*n* = 86) *n* (%)	Controls (2*n* = 240) *n* (%)	OR (95% CI)	*p*-value	Corrected *p*-value (Bonferroni method)	Power (%)
C*01	0 (0.0%)	2 (0.8%)	0.88 (0.04–18.5)	1.000	1.000	5
C*02	8 (9.3%)	21 (8.8%)	1.06 (0.46–2.41)	0.829	1.000	5
C*03	1 (1.2%)	3 (1.3%)	0.93 (0.10–8.94)	1.000	1.000	5
C*04	14 (16.3)	35 (14.6%)	1.14 (0.59–2.20)	0.726	1.000	7
C*05	4 (4.7%)	21 (8.8%)	0.51(0.17–1.53)	0.344	1.000	26
C*06	11 (12.8%)	44 (18.3%)	0.65 (0.31–1.37)	0.314	1.000	23
C*07	15 (17.4%)	64 (26.7%)	0.58 (0.30–1.11)	0.106	1.000	43
C*08	**6 (7.0%)**	**2 (0.8%)**	**8.92 (1.78**–**44.7)**	**0.005**	0.065	80
C*12	**10 (11.6%)**	**3 (1.3%)**	**10.35 (2.74**–**39.1)**	**0.0001**	0.0013	96
C*14	3 (3.5%)	7 (2.9%)	1.20 (0.30–4.80)	0.727	1.000	6
C*15	9 (10.5%)	16 (6.7%)	1.64 (0.68–3.95)	0.248	1.000	19
C*16	2 (2.5%)	11 (4.6%)	0.53 (0.11–2.56)	0.526	1.000	17
C*17	3 (3.5%)	11 (4.6%)	0.75 (0.21–2.71)	1.000	1.000	7

OR, odds ratio; CI, confidence interval, which describes the odds of cases being HLA allele carriers to the odds of controls being HLA allele carriers. Specifically, OR = 1 indicates that the factor has no impact on the occurrence of the disease, OR >1 indicates that the factor is a risk factor, and OR <1 indicates that the factor is protective.

The frequencies of the HLA-C allelic groups are displayed in **Figure 3** below, which clearly illustrates the prevalence of the HLA-C*12 and HLA-C*08 allelic groups in patients with AML compared with the control group.

### 3.6 Frequency of HLA-C genotypes in patients with AML

By analyzing different HLA-C genotypes in patients with AML and controls, we observed that C*07/C*12 was significantly more frequent in patients, with 7% as indicated in **Table 7**. The results did not include cases where the HLA-C genotype frequency was less than three in both groups.

**Table 7:** Comparative frequency of HLA-C genotypes in patients with AML and controls.

HLA-C genotypes	AML cases (*n* = 43) *n* (%)	Controls (*n* = 120) *n* (%)	OR (95% CI)	*p*-value	Corrected *p*-value (Bonferroni method)	Power (%)
C*02/C*04	2 (4.7%)	3 (2.5%)	1.93 (0.31–11.9)	0.608	1.000	8
C*02/C*05	0 (0.0%)	4 (3.3%)	0.31 (0.02–5.91)	0.574	1.000	5
C*02/C*06	0 (0.0%)	6 (5.0%)	0.20 (0.01–3.72)	0.342	1.000	12
C*02/C*07	2 (4.7%)	6 (5.0%)	0.94 (0.19–4.66)	1.000	1.000	5
C*04/C*05	0 (0.0%)	3 (2.5%)	0.41 (0.02–8.10)	0.567	1.000	5
C*04/C*06	0 (0.0%)	6 (5.0%)	0.20 (0.01–3.72)	0.342	1.000	12
C*04/C*07	3 (7.0%)	12 (10.0%)	0.68 (0.18–2.52)	0.761	1.000	8
C*05/C*06	1 (2.3%)	6 (5.0%)	0.45 (0.05–3.88)	0.677	1.000	7
C*05/C*07	0 (0.0%)	4 (3.3%)	0.31 (0.02–5.91)	0.574	1.000	5
C*06/C*07	2 (4.7%)	9 (7.5%)	0.60 (0.13–2.82)	0.729	1.000	8
C*06/C*14	0 (0.0%)	3 (2.5%)	0.41 (0.02–8.10)	0.567	1.000	5
C*06/C*15	1 (2.3%)	3 (2.5%)	0.91 (0.09–9.10)	1.000	1.000	5
C*07/C*07	1 (2.3%)	9 (7.5%)	0.29 (0.04–2.34)	0.294	1.000	18
C*07/C*12	**3 (7.0%)**	**0 (0.0%)**	21 (1.09–4.38)	**0.017**	**0.289**	**67**
C*07/C*15	2 (4.7%)	8 (6.7%)	0.69 (0.14–3.39)	1.000	1.000	9
C*07/C*17	1 (2.3%)	3 (2.5%)	0.91 (0.09–9.10)	1.000	1.000	5
C*16/C*17	1 (2.3%)	3 (2.5%)	0.91 (0.09–9.10)	1.000	1.000	5

OR, odds ratio; CI, confidence interval, which describes the odds of cases being HLA allele carriers to the odds of controls being HLA allele carriers. Specifically, OR = 1 indicates that the factor has no impact on the occurrence of the disease, OR >1 indicates that the factor is a risk factor, and OR <1 indicates that the factor is protective.

### 3.7 Frequency of HLA-C allelic groups according to AML subtypes

Based on the statistical significance of the AML subgroups, we will examine only the AML-2 subtype, given the sample size and the statistically significant results found.

#### 3.7.1 Frequency of HLA-C allelic groups in patients with AML-2

**Figure 4:**
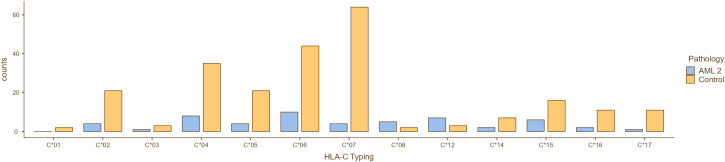
Comparative frequency of HLA-C between patients with AML-2 and controls.

We analyzed the distribution of allelic groups within the study population of 27 patients with AML-2 and 120 healthy individuals. The average age of the patients with AML-2 was 25.5 years, with a range of 10 to 41 years: Among the 13 identified HLA-C allelic groups, HLA-C*08 was the most common with 18.5%; HLA-C*12 (p = 0.0007; OR = 9.88) and HLA-C*08 (p = 0.001; OR = 12.2) show an association with AML 2 as shown in **Table 8** and **Figure 4** below.

**Table 8:** Likelihood of the frequency of the HLA-C alleles in Moroccan patients with AML-2 and controls.

HLA-C allele groups	AML 2 (2*n* = 54) *n* (%)	Controls (2*n* = 240) *n* (%)	OR (95% CI)	*p*-value	Corrected *p*-value (Bonferroni method)	Power (%)
C*01	0 (0.0%)	2 (0.8%)	0.88 (0.04–18.5)	1.000	1.000	5
C*02	4 (7.4%)	21 (8.8%)	0.82 (0.27–2.48)	0.796	1.000	6
C*03	1 (1.9%)	3 (1.3%)	1.48 (0.15–14.6)	0.581	1.000	5
C*04	8 (14.8%)	35 (14.6%)	1.01 (0.44–2.34)	1.000	1.000	5
C*05	4 (7.4%)	21 (8.8%)	0.82 (0.27–2.48)	0.796	1.000	6
C*06	10 (18.5%)	44 (18.3%)	1.01 (0.47–2.20)	1.000	1.000	5
C*07	**4 (7.4%)**	**64 (26.7%)**	0.22 (0.08–0.61)	**0.004**	**0.052**	**74**
C*08	**5 (9.3%)**	**2 (0.8%)**	12.2 (2.28–65.3)	**0.001**	**0.013**	**83**
C*12	**6 (11.11%)**	**3 (1.3%)**	9.88 (2.36–41.4)	**0.0007**	**0.0091**	**88**
C*14	2 (3.7%)	7 (2.9%)	1.29 (0.27–6.21)	0.689	1.000	6
C*15	7 (11.1%)	16 (6.7%)	1.75 (0.66–4.63)	0.174	1.000	18
C*16	2 (3.7%)	11 (4.6%)	0.80 (0.18–3.55)	1.000	1.000	8
C*17	1 (1.9%)	11 (4.6%)	0.40 (0.05–3.11)	0.472	1.000	12

OR, odds ratio; CI, confidence interval, which describes the odds of cases being HLA allele carriers to the odds of controls being HLA allele carriers. Specifically, OR = 1 indicates that the factor has no impact on the occurrence of the disease, OR >1 indicates that the factor is a risk factor, and OR <1 indicates that the factor is protective.

The frequencies of the HLA-C allelic groups are shown in **Figure 4** below, which clearly illustrates how common the HLA-C*12 and HLA-C*08 allelic groups are in patients with AML-2 compared to the control group.

#### 3.7.2 Frequency determination of HLA-C genotypes in patients with AML-2

We also analyzed HLA-C genotypes in 27 patients with AML-2 and 120 healthy individuals as a control; as a result, we did not find any association, as shown in **Table 9** below. The frequency of the HLA-C genotype less than three in both groups was not included in the results.

**Table 9:** Likelihood of the frequency of the HLA-C allele genotypes in patients with AML-2 and controls.

HLA-C genotypes	AML 2 (*n* = 27) *n* (%)	Controls (*n* = 120) *n* (%)	OR (95% CI)	*p*-value	Corrected *p*-value (Bonferroni method)	Power (%)
C*02/C*04	2 (7.4%)	3 (2.5%)	3.12 (0.49–19.7)	0.149	1.000	21
C*02/C*05	0 (0.0%)	4 (3.3%)	0.48 (0.02–9.52)	0.344	1.000	6
C*02/C*06	0 (0.0%)	6 (5.0%)	0.30 (0.02–5.46)	0.199	1.000	9
C*02/C*07	2 (7.4%)	6 (5.0%)	1.52 (0.28–8.10)	0.257	1.000	10
C*04/C*05	0 (0.0%)	3 (2.5%)	0.65 (0.03–13.5)	0.412	1.000	5
C*04/C*06	0 (0.0%)	6 (5.0%)	0.30 (0.02–5.46)	0.199	1.000	9
C*04/C*07	0 (0.0%)	12 (10.0%)	0.14 (0.01–2.43)	0.094	1.000	27
C*05/C*06	1 (3.7%)	6 (5.0%)	0.73 (0.08–6.44)	0.297	1.000	6
C*05/C*07	0 (0.0%)	4 (3.3%)	0.48 (0.02–9.52)	0.344	1.000	6
C*06/C*07	1 (3.7%)	9 (7.5%)	0.47 (0.06–3.74)	0.217	1.000	8
C*06/C*14	0 (0.0%)	3 (2.5%)	0.65 (0.03–13.5)	0.412	1.000	5
C*06/C*15	1 (3.7%)	3 (2.5%)	1.52 (0.15–15.4)	0.374	1.000	7
C*06/C*16	1 (3.7%)	5 (4.2%)	0.88 (0.09–8.49)	0.305	1.000	5
C*07/C*07	0 (0.0%)	9 (7.5%)	0.20 (0.01–3.47)	0.153	1.000	18
C*07/C*15	1 (3.7%)	8 (6.7%)	0.53 (0.06–4.59)	0.247	1.000	7
C*07/C*17	0 (0.0%)	3 (2.5%)	0.65 (0.03–13.5)	0.412	1.000	5
C*16/C*17	1 (3.7%)	3 (2.5%)	1.52 (0.15–15.4)	0.374	1.000	7

OR, odds ratio; CI, confidence interval, which describes the odds of cases being HLA allele carriers to the odds of controls being HLA allele carriers. Specifically, OR = 1 indicates that the factor has no impact on the occurrence of the disease, OR >1 indicates that the factor is a risk factor, and OR <1 indicates that the factor is protective.

## 4 Discussion

In this case–control study, BCP-ALL constituted the majority of ALL cases, comprising 75.43% of patients. A notable rise in the frequencies of the HLA-C12 and HLA-C08 alleles, along with the C05/C12 genotype, was detected in patients with ALL. Conversely, none of the analyzed HLA-C genotypes shown either susceptibility or protective influence. Analogous trends were noted in the BCP-ALL subgroup, characterized by increased frequencies of HLA-C*12.

In patients diagnosed with AML, the AML-2 subtype was the most common, representing 62.79% of cases. Notable correlations were found between AML and the HLA-C12 and HLA-C08 alleles, with similar findings obtained for the AML-2 subtype. The findings align with other reports indicating that polymorphisms in HLA class I genes may affect leukemia susceptibility, although the specific alleles linked to illness risk may vary among cultures and research.

Preliminary evidence associating HLA antigens with leukemia susceptibility originated from extensive case–control studies indicating that specific HLA-C antigens, notably Cw3 and Cw4, were more prevalent in patients with ALL and AML compared to healthy controls. The findings indicated that genetic variation in the HLA-C locus may play a role in leukemogenesis or affect the immunological identification of leukemic cells. The research revealed significantly higher frequencies of Cw3 and Cw4 in patients with AL, while no significant relationships were found for other HLA antigens after adjusting for multiple comparisons. This pattern indicates the potential for allele-specific effects in the HLA-C area concerning leukemia susceptibility (13).

Recent investigations have underscored the significance of HLA class I molecules in particular AML subtypes distinguished by unique molecular aberrations. In individuals with AML possessing a nucleophosmin 1 (NPM1) mutation, specific HLA class I alleles, including HLA-C*07:01, were seen to be less prevalent. Furthermore, the existence of these alleles correlated with enhanced survival, indicating that particular HLA genotypes may affect both illness susceptibility and prognosis (14).

In addition to AL, several HLA-C alleles have been linked to hematological malignancies in diverse situations, suggesting locus-wide immunogenetic effects. HLA-C3 was linked to an elevated risk in a Korean cohort of patients with AML, encompassing particular disease subgroups and clinical outcomes (15). Nonetheless, many HLA-C alleles, aside from leukemia, have been associated with other hematologic malignancies, including multiple myeloma and diffuse large B-cell lymphoma, highlighting the extensive significance of HLA class I variation in hematological cancers and immune surveillance.

A study in a Turkish population identified HLA-C07 (*p* = 0.01) and HLA-C06 (*p* = 0.03) as protective alleles against leukemia (11). Correspondingly, a study conducted in a Chinese population found HLA-C*06 as a protective allele in individuals with ALL (16). An Indian investigation indicated that HLA-C*07 was linked to a heightened risk of AML (*p* = 0.04) (17). These data underscore the heterogeneity of HLA-C correlations among diverse groups.

Comparative analysis with prior studies indicates both congruities and disparities in HLA-C correlations with leukemia. Preliminary studies in the Netherlands indicated a correlation between leukemia and HLA-C antigens, specifically Cw3 and Cw4 (15). A study from Germany similarly revealed a correlation between HLA-C*03 and ALL (18). This observation is additionally corroborated by a Korean study that revealed a correlation between HLA-C*03 and AML. The fundamental process may entail the capacity of specific HLA-C molecules to influence NK cell-mediated immune surveillance, thereby diminishing the immune system’s efficacy in identifying and eradicating leukemic cells, therefore facilitating leukemia progression (15).

Recent studies have corroborated the involvement of HLA class I polymorphisms in leukemia susceptibility and immune surveillance. In a substantial European cohort, specific HLA class I alleles, notably HLA-C*03:04, were significantly underrepresented in patients with NPM1-mutated AML, suggesting a protective effect via enhanced anti-leukemic immune responses. The observations support the notion that certain HLA-C alleles may influence the recognition and elimination of leukemic cells by cytotoxic T lymphocytes (19). A recent analysis of the prevalence of HLA alleles in patients with AL revealed a notable disparity in HLA distribution between these patients and healthy controls, thereby reinforcing the association of HLA polymorphisms with disease susceptibility. Nonetheless, the particular alleles associated with risk or protection varied among groups, indicating that ethnic and regional genetic origins significantly affect HLA–disease relationships (11). The findings of the current study align with this population-specific trend. In our Moroccan sample, HLA-C12 and HLA-C08 were associated with increased susceptibility, but HLA-C*07 showed a potential protective effect in certain subgroup analyses; however, these associations differ from those reported in European populations. Such disparities are anticipated, given the extensive genetic diversity of the HLA area and the significant variations in allele frequencies among ethnic groupss (20). Collectively, our data suggest that HLA-C polymorphisms’ influence on leukemia susceptibility is likely affected by the genetic background of the population and may indicate alterations in antigen presentation and immune surveillance. Replication studies in larger and ethnically diverse cohorts are necessary to validate these associations and to clarify their biological significances (21).

Our results concerning HLA-C08 and HLA-C12 indicate that the influence of particular alleles may vary according to ethnic background, sample size, and case definition. This conclusion aligns with prior studies indicating variability in HLA–disease relationships among diverse geographic locations and people. Differences in allele frequencies and linkage disequilibrium patterns among populations may account for these disparities. Thus, genes that enhance disease susceptibility in one population may seem neutral or even advantageous in another, highlighting the necessity of performing genetic association studies in varied and well-defined cohorts.

This study’s observed genetic relationships lack corresponding reports in the literature, hindering direct comparison with existing findings.

This study has certain limitations that must be acknowledged. The limited sample size, particularly for the AML-M2 and BCP-ALL subgroups, may have affected the accuracy of the calculated ORs, resulting in wide CIs for different alleles and genotypes. *Post-hoc* power analyses were performed, and Bonferroni correction was applied to reduce the probability of false-positive results; however, larger studies are required to validate the observed associations. The cross-sectional case–control methodology can only demonstrate the statistical association between HLA-C polymorphisms and susceptibility or protection against AL, but it cannot establish causality effect, which requires longitudinal and functional studies aiming to clarify the biological mechanisms of the observed connections. This study was conducted within a specific Moroccan population, and the genetic background of the participants may vary from that of other regions. Consequently, the present findings may not be easily applicable to different ethnic or geographic areas. Another limitation of this study is the absence of accurate clinical and hematological data, including hemoglobin levels, WBC counts, platelet counts, and bone marrow blast percentages. This information was not systematically accessible, as the investigation was conducted in a specialized HLA histocompatibility laboratory primarily focused on HLA typing.

## 5 Conclusion

This study provides new information regarding the association between the susceptibility of the Moroccan population to AL and HLA-C polymorphisms. Our findings underscore the potential significance of immunogenetic variables in the etiology of leukemia, indicating that particular HLA-C alleles may be associated with heightened vulnerability or protection against disease onset. Importantly, even after correcting for multiple tests, HLA-C*12 remained a significant susceptibility marker. These constatations could have clinical implications that go beyond their biological relevance. HLA-C polymorphisms may be utilized in future risk stratification models and could function as potential genetic markers for identifying individuals with heightened susceptibility to AL. Moreover, an enhanced comprehension of the immunological mechanisms governed by HLA may facilitate the formulation of customized treatment strategies, including immunotherapy, and the optimization of donor selection in HSCT. However, such data require further validation in larger and more diverse ethnic populations.

## Institutional review board statement

Institutional Review Board Statement: The Research Ethics Committee (REC) of the Faculty of Medicine and Pharmacy at Cadi Ayyad University, Marrakech, Morocco. Approval Code: 34/2022. Approval date: 27 February 2023. Informed Consent Statement: The samples analyzed in this study were obtained as a part of the routine activities of the HLA laboratory of Mohammed VI University Hospital in Marrakech.

## Informed consent statement

Sociodemographic data were extracted from the computer database anonymously under the supervision of the laboratory manager. In this case, approval of ethics and informed consent were not necessary.

## Data Availability

The data used in this study are not publicly available due to privacy restrictions. Access to the data is restricted in accordance with the ethical guidelines and regulations governing the protection of participant confidentiality and privacy. However, researchers interested in replicating or verifying the findings presented in this study may request access to the data through the appropriate institutional review board. Requests for data access will be considered on a case-by case basis, subject to approval by the relevant authorities and compliance with applicable privacy regulations. For inquiries regarding data access, please contact Pr. Brahim ADMOU/Clinical research Center/Mohammed VI University Hospital Center at br.admou@uca.ac.ma.
